# Gut microbiota and epigenetic choreography: Implications for human health: A review

**DOI:** 10.1097/MD.0000000000039051

**Published:** 2024-07-19

**Authors:** Bailee Kim, Angel Song, Andrew Son, Yonghwan Shin

**Affiliations:** a Crescenta Valley High School, La Crescenta, CA; b Harvard-Westlake School, Studio City, CA; c Bellarmine College Preparatory, San Jose, CA; d Department of Biochemistry and Molecular Medicine, Norris Comprehensive Cancer Center, University of Southern California, Los Angeles, CA.

**Keywords:** DNA methylation, epigenetic, gut microbiota, histone modification

## Abstract

The interwoven relationship between gut microbiota and the epigenetic landscape constitutes a pivotal axis in understanding human health and disease. Governed by a myriad of dietary, genetic, and environmental influences, the gut microbiota orchestrates a sophisticated metabolic interplay, shaping nutrient utilization, immune responses, and defenses against pathogens. Recent strides in genomics and metabolomics have shed light on the intricate connections between these microbial influencers and the host’s physiological dynamics, presenting a dynamic panorama across diverse disease spectra. DNA methylation and histone modifications, as key players in epigenetics, intricately align with the dynamic orchestration of the gut microbiota. This seamless collaboration, notably evident in conditions like inflammatory bowel disease and obesity, has captured the attention of researchers, prompting an exploration of its nuanced choreography. Nevertheless, challenges abound. Analyzing data is intricate due to the multifaceted nature of the gut microbiota and the limitations of current analytical methods. This underscores the need for a multidisciplinary approach, where diverse disciplines converge to pave innovative research pathways. The integration of insights from microbiome and epigenome studies assumes paramount importance in unraveling the complexities of this intricate partnership. Deciphering the synchronized interactions within this collaboration offers a deeper understanding of these delicate interplays, potentially heralding revolutionary strides in treatment modalities and strategies for enhancing public health.

## 1. Introduction

The intricate relationship between human health and the gut microbiota serves as a cornerstone in understanding essential functions such as digestion, vitamin synthesis, and immune regulation. This dynamic symbiosis is continually shaped by various factors including dietary choices, genetic makeup, age, and environmental influences. Notably, its composition and functionality undergo significant alterations during disease progression and early developmental phases.^[1]^ The interplay between the microbiota, dietary patterns, and environmental factors is pivotal in the onset of chronic illnesses,^[2]^ positioning it as a central player in both human and microbial wellness.^[3]^ Given its pervasive involvement in host physiological processes and disease mechanisms, the gut microbiota has emerged as a critical target for healthcare interventions.^[4]^ The study of the gut microbiota has undergone a substantial evolution. Early techniques reliant on culture-dependent methods provided limited insights. However, the advent of high-throughput sequencing technologies has revolutionized this field, unveiling the complex and diverse microbial populations within the human gut. This diversity plays an integral role in various health-related processes. Despite challenges posed by individual variability and temporal fluctuations, which complicate clinical applications, the gut microbiota is increasingly acknowledged as a vital “organ” with a profound influence on health and disease. The intricate interconnection between gut microbiota and host metabolic activities stands as a crucial area of study within epigenetics.^[5,6]^ This association is particularly evident in conditions such as inflammatory bowel disease (IBD), where epigenetic modifications play pivotal roles in disease progression.^[7]^ These modifications, spanning DNA methylation, histone modification, and the roles of noncoding RNA, are significantly influenced by environmental shifts and the gut microbiota. This highlights the dynamic and adaptable nature of epigenetic changes.^[8–10]^ The foundational relationship between gut microbiota and epigenetic factors profoundly impacts human health and disease prevention. This interplay orchestrates numerous biological processes, encompassing intestinal inflammation, metabolic imbalances, and colitis incidents. With a vital role in both metabolic and immune functions, the gut microbiota significantly correlates with chronic health conditions. A comprehensive understanding of the gut microbiota, particularly its interactive dynamics with host tissues, is pivotal in developing predictive models for disease diagnostics and therapeutic strategies. The impact of the gut microbiota on standard host physiological processes and its involvement in diverse diseases underscores its indispensable role in maintaining health and influencing disease emergence.

## 2. Methods

### 2.1. Search strategy

To ensure a comprehensive review, we conducted a thorough literature search using PubMed, Scopus, and Web of Science databases. The search terms included “gut microbiota,” “epigenetics,” “infectious diseases,” “DNA methylation,” “histone modification,” “inflammatory bowel disease,” and “obesity.” The search was limited to articles in English and included studies involving both human subjects and animal models that provide mechanistic insights.

### 2.2. Inclusion and exclusion criteria

We selected peer-reviewed studies involving human subjects, animal studies providing mechanistic insights relevant to gut microbiota and epigenetics, articles addressing the impact of gut microbiota on diseases such as IBD, obesity, and neurological disorders, and studies discussing the molecular mechanisms of DNA methylation and histone modification influenced by gut microbiota. We aimed to include studies with various designs, such as observational studies, experimental studies, and clinical trials, covering a range of cutting-edge technologies, for example, high-throughput sequencing, epigenetic profiling, and metabolomic analysis. We excluded non-peer-reviewed articles, studies not specifically focused on the interaction between gut microbiota and epigenetics, articles not available in English, and studies with insufficient or unclear methodologies.

### 2.3. Sample sizes

Sample sizes of the reviewed studies vary greatly, for instance, a small group of individual studies (n < 50) but great detail in the mechanistic insight and poor generalization, and large population studies (n > 500) with high statistical power and high applicability of findings. Experimental animal studies are conducted with 10 to 30 subjects per experimental group, which makes the conditions under controlled experimental conditions.

### 2.4. Data extraction and synthesis

A search of the databases of PubMed, Scopus, and Web of Science was done to find the relevant articles. Screening of the titles and abstracts of articles was done. Full-text articles of potentially relevant studies were obtained. Sample size, study design, key findings, and conclusions were extracted. The extracted data was synthesized for the combined review of the gut microbiota and its effect on epigenetic mechanisms. Information was then synthesized into themes and findings.

### 2.5. Limitation of the current review

*Database and language restrictions:* This review considered only studies that were in the English language and sourced from specific databases - PubMed, Scopus, and Web of Science. This might lead to the exclusion of relevant studies published in other languages or indexed in different databases and potentially bias the results. *Study selection criteria*: Inclusion criteria restrict the review to the design of human study, mainly peer-reviewed articles that concern human subjects and relevant animal models. Such a limitation may introduce selection bias by excluding some articles that are likely to be published with adverse findings and are also prone to miss gray literature and studies not published in peer-reviewed journals, which can provide further insights. *Heterogeneity of studies*: Studies differ substantially in the study design, sample size, methodologies, and population characteristics. That makes it very difficult to attain decisive conclusions and can lead to interpretation bias when synthesizing across divergent studies. *Publication bias*: There is the possibility of publication bias because studies with significant or positive results are published and included in the review, which, in turn, would provide overestimated effects and relationships between gut microbiota, epigenetics, and health outcomes. *Variability in methodologies*: The studies vary in the methodologies of the assessment of the gut microbiota and the epigenetic modifications. Some have used different sequencing techniques, and some have used different epigenetic assays, which in turn could impact the comparability of results and the overall synthesis of findings. *Longitudinal versus cross-sectional design*: Many of the studies included in this article are cross-sectional; thus, they only provide a snapshot of associations at a single point in time. Longitudinal studies, which trace changes over time, are less well represented but are important in understanding the nature of causal relationships and long-term effects. *Generalizability*: The findings from the respective studies selected for review have low generalizability, given that most are within particular cohorts with special characteristics. The generalizability of their results might be poor. *Emerging areas of research*: Some aspects, such as the involvement of gut microbiota in neurological health through epigenetic mechanisms, have not been well established with solid evidence. The inferences made in such areas should be used while making interpretations with caution.

## 3. Gut microbiota: composition and functions

### 3.1. Introduction to gut microbiota

The human gut microbiota significantly influences disease development through nutrient metabolism, pathogen defense, and immune response modulation.^[11]^ The diversity, stability, and resilience of the gut microbiota are fundamental in preserving health.^[1]^ The microbiota’s involvement in human metabolism, encompassing digestion, metabolite production, and immune system regulation, is significant.^[12]^ Progress in gut microbiota research has been transformative. Initial studies sought to catalog the microbial inhabitants and their potential implications for human health and disease. Contemporary research, propelled by genomic and metabolomic advancements, explores the intricate symbiosis between the gut microbiota and host physiology, unraveling the ramifications of these interactions in health and disease contexts.^[13]^

### 3.2. Microbial diversity and composition

A dominance of Firmicutes from 25.3% to 67.3% and Bacteroidetes from 4.1% to 63.5% in the gut microbiota of healthy Chilean subjects was found using 16s rRNA gene sequencing. Verrucomicrobia, 8.5% to 10.4%, and Actinobacteria, 1.8% to 3.9% of our gut microbiota, also comprise a significant part of a healthy gut microbiota.^[14]^ While bacteria dominate, the human gut environment also includes archaea, viruses, fungi, and protozoa, each contributing uniquely to gut functionality.^[14]^ The gut microbiota’s composition is a barometer of gut health shaped by diet, age, medication, and other factors.^[15]^ However, gut microbiota’s intricacies and individual differences make this balance highly variable.^[16]^ The microbiota exhibits spatial diversity along the gastrointestinal tract, influenced by pH, oxygen levels, and nutrient access. These factors selectively foster microorganisms well-suited to the local environments, creating a richly diverse and specialized microbial population along the gastrointestinal pathway.^[17]^

### 3.3. Influences on gut microbiota composition and function

Diet, as a critical determinant, actively reshapes the nutrient spectrum available to intestinal microbes, thus profoundly influencing their collective structure. Pharmaceuticals, notably antibiotics, induce significant variations in the microbiome, often leading to a marked decrease in its diversity. Aging introduces specific shifts in the microbiota composition associated with diverse health outcomes, particularly in metabolic and immune system disorders. This complex interaction is sculpted by external influences and the host’s genetic constitution and nutritional state. The implication is that this complex connection spans a variety of bodily functions, including methods of nutrient uptake, metabolic pathways, the development of intestinal epithelial cells, and the modulation of the immune system.^[18–21]^ Due to temporal restriction feeding, subjects under dim light at night conditions and constant darkness have significantly reduced percentages of oscillating gut microbes. In a control group, MKO and DTA mice subjects have significant differences in alpha and beta gut microbe diversity compared to the control. Their gut microbiota content was similar when exposed to aberrant light. However, the abundance of each microbe significantly differed under aberrant light exposure but not in the control group.^[22]^

### 3.4. Functional capacities of gut microbiota

The gut microbiota, a cornerstone in the landscape of nutrient metabolism, plays a vital role in synthesizing vitamins and extracting energy from dietary components. Its influence pervades the metabolism of bile acids, sterols, and xenobiotics, critically impacting the host’s metabolic balance and energy homeostasis.^[23,24]^ As an integral element of the host’s defensive arsenal, the gut microbiota is crucial in maintaining the integrity of the gut barrier and orchestrating the immune response, thereby safeguarding against pathogenic invasion. The equilibrium within microbial communities is essential in averting the colonization and proliferation of pathogenic entities.^[25]^ The gut-brain axis connects the gut microbiota to the central nervous system, influencing brain function and behavior. This maintains the core information while reducing wordiness. This connection has implications for various neuropsychiatric disorders, including depression, anxiety, Parkinson disease, and autism. Microbiota-brain interactions are facilitated through neural, endocrine, and immune pathways.^[26,27]^

### 3.5. Gut microbiota and immune system dynamics

The gut microbiota’s role in immune system maturation is pivotal, orchestrating adaptive immune responses and establishing an equilibrium in immune reactions. This influence is indispensable for maintaining immune homeostasis. Through interactions with immune cells, the microbiota modulates immune responses, finely balancing pro-inflammatory and anti-inflammatory actions. This modulation critically influences systemic immune dynamics, contributing to the development and sustenance of the immune system. Notably, the microbiota’s regulatory impact on effector and regulatory T cells is essential for preserving homeostasis and mounting effective pathogen responses.^[28–30]^ Dysbiosis, typified by an imbalance in gut microbiota, is intricately linked to various immune-related diseases. Changes in the gut microbiota composition are implicated in autoimmune responses, as evident in conditions like rheumatoid arthritis, possibly through mechanisms such as molecular mimicry and activation of the immune system. Moreover, the pivotal role of gut microbiota in inflammatory/autoimmune diseases, allergies, and cancer is increasingly recognized, highlighting its integral role in these diverse yet interrelated pathological contexts.^[31,32]^ The human gut microbiota harbors diverse microbial entities and exhibits unique variability across individuals. This microbiome exerts a profound influence on a multitude of physiological functions and disease pathways. Its intricacy is deeply entwined with the host’s genetic framework, environmental exposures, and lifestyle choices, markedly influencing conditions like inflammation and obesity.^[1,33]^ Spanning the entirety of human life, the gut microbiota composition is subject to substantial metamorphosis. This dynamic evolution is critically shaped by variables such as dietary patterns, environmental interactions, and the host’s physiological status. The developmental trajectory of gut microbiota throughout varying life stages, intricately modulated by dietary and environmental factors, has been firmly established. This complex interdependence between dietary habits and microbial diversity in the gut highlights the imperative for tailored nutritional strategies, which are pivotal in sustaining health and strategically managing disease conditions.^[34,35]^

### 3.6. Therapeutic implications

The probiotics and prebiotics, pivotal in modulating gut microbiota, have garnered significant attention in recent scientific discourse. Probiotics, defined as live microorganisms conferring health benefits to the host, and prebiotics, nondigestible food components that foster the growth or activity of beneficial gut bacteria, are increasingly lauded for their role in sustaining gut health and addressing a spectrum of gastrointestinal ailments. The deployment of probiotics has been evidenced to modify gut microbiota, potentially yielding health advantages by impeding pathogenic organism colonization and averting maladaptive immune reactions.^[36]^ Fecal Microbiota Transplantation (FMT) transfers fecal matter from a healthy donor to a recipient to restore a balanced microbiota ecosystem. FMT has exhibited substantial clinical efficacy in combating Clostridium difficile infection, and bears promise in the management of other conditions linked to alterations in gut microbiota, including IBD, obesity, metabolic syndrome, and functional gastrointestinal disorders.^[37,38]^ The paradigm of personalized nutrition, which involves the customization of dietary regimens based on individual microbiota profiles, is gaining momentum. This innovative approach acknowledges each individual’s distinct gut microbiota composition and its consequential impact on health and disease states. Strategies under the umbrella of personalized nutrition endeavor to refine gut microbiota composition, thereby enhancing overall health and aiding in the prevention or management of diseases.^[39,40]^ There is an imperative for more comprehensive research into the long-standing effects of probiotics, prebiotics, and FMT and in crafting personalized nutrition plans based on microbiota profiles. Grasping these connections is fundamental to developing more advanced therapeutic strategies for diverse health conditions.

## 4. Epigenetics: basics and mechanisms

### 4.1. Introduction to epigenetics

Epigenetic regulation, a foundational element of genetic function, surpasses the scope of DNA sequencing. It encompasses a wide spectrum of processes, notably encompassing DNA methylation, alterations in histone proteins, and the intricate workings of noncoding RNAs (ncRNAs). These mechanisms collectively oversee a multitude of biological processes and pathways that influence disease development. Recent advancements in this field underscore its paramount importance in unraveling gene regulatory mechanisms and their consequential roles in both health and disease contexts. Within this dynamic and evolving landscape, the study of epigenetics provides profound insights into the nuanced control of gene expression and its extensive implications.^[41]^

### 4.2. Fundamental epigenetic mechanisms

DNA methylation, a pivotal epigenetic control mechanism, involves adding methyl groups to DNA, predominantly at cytosine bases. This process is vital for controlling gene activity. DNA methylation’s role is pronounced in various diseases, underscoring its relevance in healthy physiology and pathological states. These epigenetic elements, including their interplay in cancer development, significantly shape gene expression patterns.^[42–44]^ Alterations to histones, the proteins around DNA coils, constitute another cornerstone of epigenetic regulation. These modifications, spanning acetylation, methylation, phosphorylation, and ubiquitination, modulate chromatin architecture, thereby influencing gene expression. The fluid nature of histone modifications is instrumental in modulating gene activity and expression, shaping the cell’s epigenetic terrain.^[45,46]^ Noncoding RNAs, including miRNAs and lncRNAs, are central to posttranscriptional gene regulation. These entities participate in various functions, from RNA silencing to gene expression modulation, and are acknowledged for their roles in the epigenetic regulatory framework. Recent studies emphasize the significance of ncRNAs in the epigenetic regulatory network, highlighting the intricate and crucial nature of epigenetic mechanisms in gene regulation.^[47]^

### 4.3. Epigenetics in development and aging

Epigenetic dynamics are pivotal in embryonic development and cellular differentiation. Regulation of chromatin architecture plays a crucial role in modulating gene activation or repression, thereby influencing cellular identity and the structural organization of the nucleus.^[48]^ In aging, these modifications impact gene expression and cellular function. Over time, variations in DNA methylation influence the onset and progression of age-related diseases. An example is the escalated activity of H3K27 demethylase Utx-1 during aging, which contributes to a gradual increase in gene expression and a corresponding decrease in H3K27me3 on insulin/IGF-1 signaling components. The phenomenon of stochastic DNA methylation drift in aging cells leads to the development of epigenetic mosaicism. This alteration has the potential to limit cellular plasticity and exacerbate phenotypes such as stem cell exhaustion and localized proliferative anomalies, which might escalate the risk of cancer development.^[49]^

### 4.4. Epigenetic dynamics: The interplay in health and disease

Epigenetic landscapes, characterized by mechanisms like DNA methylation, histone modification, and noncoding RNA involvement, are fundamental in various health conditions and diseases. In cancer, epigenetic alterations can lead to the activation of oncogenes or the suppression of tumor suppressor genes. Such shifts, impacting neurological disorders like Alzheimer, influence disease progression.^[50,51]^ Metabolic diseases, including obesity and type 2 diabetes, are similarly impacted by epigenetic variations that dictate gene expression patterns.^[52]^ The profound influence of environmental elements on epigenetic configurations has been extensively documented.^[53]^ Further, the interaction between dietary polyphenols and epigenetic mechanisms has been explored, revealing potential chemopreventive actions in cancer.^[54]^

### 4.5. Advancements in epigenetic technology landscape

Recent technological breakthroughs have transformed the landscape of epigenetic research. The advent of Next-Generation Sequencing technologies has enabled an extensive mapping of epigenetic alterations across genomes. Development of the CRISPR-Cas9 technology has been a notable breakthrough as this innovative technology has allowed researchers to carry out precise epigenome editing. CRISPR-Cas9 enables targeted modifications in DNA methylation patterns and histone configurations and also presents a robust framework for investigative and potential therapeutic interventions to repair epigenetic irregularities.^[55]^

### 4.6. Relationship between gut microbiota and epigenetics

Studies are rapidly advancing our understanding of how gut microbiota and host epigenetic mechanisms interact to maintain health and prevent disease. Gut microbiota and epigenetic modifications mutually influence essential gene expression areas, including immune responses, metabolic activities, and neurological health.^[56,57]^ This synergy is crucial for understanding complex diseases. Gut microbiota’s ability to modulate the host’s epigenetics offers new insights into the foundations of health disorders, from metabolic syndromes to neurological conditions.

### 4.7. Mechanisms of microbiota-induced epigenetic modifications

Gut microbiota elements, especially bacterial byproducts, drive epigenetic modifications in host cells. Short-chain fatty acids (SCFAs), a bacterial fermentation byproduct of dietary fibers, affect DNA methylation and histone modifications. The pathways through which these metabolites modulate epigenetic states entail intricate interactions with the host’s cellular systems. SCFAs can modify histone acetylation, thus affecting gene transcription. They can also adjust DNA methylation patterns, culminating in altered gene expression landscapes.^[58]^ The epigenetic transformations instigated by gut microbiota metabolites bear significant consequences for the host’s health. These changes govern genes central to immune reactions, metabolic routes, and neural functions. This underscores the profound influence of the connection between gut microbiota epigenetics and the host’s overall health state and disease vulnerability.^[59]^

### 4.8. Epigenetic regulation of gut microbiota

The interplay between the host’s epigenetic mechanisms, especially histone acetylation and deacetylation, is critical in the gut microbiota-host relationship. Dietary metabolites from the gut microbiota are central to this regulation. These metabolites serve as key modulators, instigating epigenetic alterations that shape the composition of the gut microbiota.^[60,61]^ The area of noncoding RNAs, with microRNAs at the forefront, emerges as significant contributors from the host, steering the gut microbiome’s landscape. These microRNAs help maintain gut microbiota equilibrium. Intriguingly, they are susceptible to modifications prompted by the host’s epigenetic framework shifts. Such intricate crosstalk is critical in a spectrum of health contexts, extending to mental health disorders like depression. In these scenarios, metabolites from the gut microbiome trigger epigenetic shifts.^[62]^ Host epigenetic modifications can precipitate a state of dysbiosis marked by an imbalance in the gut microbiota. This dysbiotic state is intricately linked with various health ramifications, notably influencing brain function and behavioral patterns. Products from gut microbes can modulate chromatin architecture within the host’s brain. This modulation, in turn, precipitates alterations in neuronal transcription and subsequent behavioral changes.^[63]^

### 4.9. Disease associations and clinical implications

The relationship between gut microbiota and epigenetic mechanisms plays a key role in diseases like IBD, obesity, and several mental disorders, such as autism, bipolar disorder, schizophrenia, and major depressive disorder.^[64]^ Exploring the gut microbiota-epigenetics connection offers potential for therapeutic advances. The strategic use of specific probiotics or dietary elements, known to modulate the microbiome and epigenome concurrently, offers a novel approach to treating or managing diseases linked to this axis. The dynamic interplay between the microbiome and the host’s central nervous system, particularly its implications in managing obesity and cardiometabolic diseases, remains an active field of investigation.^[65]^

### 4.10. Environmental influences on the gut microbiota–epigenetics axis

Environmental factors such as diet, stress, and toxin exposure significantly shape the gut microbiota–epigenetics connection. Dietary components, for instance, can remodel gut microbial communities, engendering metabolites that exert epigenetic influences on host cells. Stress and toxins similarly impinge on this axis, modulating gene expression, phenotypic outcomes, and disease evolution.^[53,66]^ The interplay of environmental factors, gut microbiota, and epigenetics is crucial in developing various diseases. Environmental shifts impacting the gut microbiota–epigenetics axis may lead to altered immune reactions, metabolic imbalance, and neurological function changes, thereby accelerating disease progression.^[1,67,68]^

### 4.11. Gut microbiota, epigenetics, and infectious diseases

The interplay between gut microbiota, epigenetic mechanisms, and infectious agents is decisive in disease progression and clinical outcomes. This includes influencing the balance between Th17 cells and regulatory T cells (Treg), key regulators of inflammatory and metabolic diseases. Research has shown that these relationships are crucial in the body’s defense against pathogens, emphasizing the need for deeper understanding.^[69]^ In the complex area of infectious diseases, the intersection of gut microbiota and epigenetic mechanisms is a critical determinant. This triad of interactions, which includes the microbiota, epigenetic changes, and infectious agents, significantly impacts the host’s defensive mechanisms against pathogens. The interactions between various biological mechanisms are integral in steering the progression of diseases and molding clinical outcomes, highlighting the imperative for a deepened grasp of these interactions.

### 4.12. Influencing disease susceptibility

Changes in gut microbiota composition and epigenetic patterns influence individual vulnerability to infectious diseases. Crucial epigenetic processes, notably DNA methylation and chromatin modification, largely influenced by environmental elements, are central in dictating susceptibility to diseases. Factors such as microbial imbalance and epigenetic modifications can serve as precursors, amplifying the risk of infections. For example, dietary patterns that disrupt gut microbiota balance have been linked to elevated risks of IBD and other infectious diseases. How the gut microbiota impacts epigenetic modifications within the immune system can significantly alter an individual’s susceptibility to infectious diseases.^[59,70]^

### 4.13. Influence of gut microbiota and epigenetics on infectious diseases

The gut microbiota is instrumental in driving epigenetic transformations fundamental to the progression of infectious diseases. This elaborate microbial network subtly influences the immune system’s epigenetic configuration, primarily leveraging DNA methylation and histone modification as conduits to alter the course of infectious agents.^[71]^ Such epigenetic transitions, critical in their breadth and influence, are key determinants in shaping the intensity, progression, and final ramifications of a diverse spectrum of infectious diseases. With their far-reaching implications, these epigenetic modifications profoundly impact the severity, course, and eventual outcomes of various infectious diseases (Table 1).

**Table 1:** Examples of specific infectious diseases.

Infectious disease	Gut microbiota influence	Epigenetic influence	References
Bacterial infections	Influenced through bacterial metabolism, stimulation of host immunity, and direct bacterial antagonism.	Epigenetic marks allow microbial persistence or play a role in “hit-and-run” infectious mechanisms.	Leslie and Young (2015); Barros et al (2018)^[72,73]^
Inflammatory Bowel Disease (IBD)	Disturbances in gut bacterial microbiota may underlie disorders like IBD, potentially controlling the immune system.	Epigenetic events influence cytokine production, contributing to inflammatory diseases like IBD.	Round and Mazmanian (2009); Gomez et al (2009)^[74,75]^
Necrotizing Enterocolitis (NEC) in Preterm Infants	Gut microbiota may predispose preterm infants to NEC due to factors like gestational age, mode of delivery, and diet.	Epigenetic processes are key in how the early environment influences cell function and metabolism, affecting diseases like NEC.	Collado et al (2015); Fernandez-Twinn et al (2015)^[76,77]^
Periodontal Diseases	Gut microbiota influences susceptibility to diseases like periodontitis through interactions with the immune system.	Complex epigenetic crosstalk involving environmental influences affects the pathogenesis of periodontal diseases.	Mukherjee et al (2018); Barros and Offenbacher (2014)^[78,79]^
Infectious Keratitis	Gut microbiota plays a role in influencing lung diseases and respiratory infections, which can impact conditions like keratitis.	Epigenetic mechanisms influence the pathogenesis of complex eye diseases like infectious keratitis.	Dhar and Mohanty (2020); Verma et al (2021)^[80,81]^
Gastric Cancer	Alterations in gut microbiota can influence the susceptibility to diseases like gastric cancer.	Epigenetic alterations (DNA methylation) influence premalignant lesions of cancer at early stages.	Cani (2018); Corvalán and Maturana (2016)^[82,83]^
Cardiovascular Disease	Gut microbiota composition is influenced by environmental factors, affecting diseases like cardiovascular disease.	Epigenetics may partially explain the complex nature of cardiovascular disease.	Cani (2018); Corwin (2004)^[82,84]^
Lung Cancer	Gut microbiota plays a role in influencing lung diseases, which can include lung cancer.	Epigenetic marks influence immune cell maturation, associated with various forms of cancer, including lung cancer.	Dhar and Mohanty (2020); Yang and Schwartz (2011)^[80,85]^

IBD = inflammatory bowel disease.

### 4.14. Pathogen–host interactions

The dynamic interplay between pathogens and the gut microbiota shapes host epigenetic regulation. The gut microbiota influences bacterial infections through a spectrum of mechanisms, encompassing bacterial metabolism, the stimulation of host immune responses, and direct antagonistic actions against pathogens. These interactions are pivotal in refining our comprehension of infectious diseases and the host’s reaction to these challenges.^[72]^ The gut microbiota–host axis emerges as a fundamental element in health sustenance, with epigenetic mechanisms such as DNA methylation and histone modifications playing instrumental roles in this intricate relationship.^[56]^ Pathogens can elicit profound alterations in the composition of the gut microbiota. This shift, in turn, can cascade into modifications within the host’s epigenetic framework. Such transformations are pivotal in shaping disease presentation and the efficacy of host defense strategies. A notable instance is gut microbiota’s modulation of transcriptional dynamics in human colonic epithelial cells. This modulation is influenced by the host’s genetic makeup, shedding light on the intricacies of host–microbiota interplay. These insights are crucial in understanding obesity and colorectal cancer.^[86]^ This intersection of microbial activity and host genetic context underscores the complexity and significance of these interactions in disease pathogenesis and progression.

### 4.15. Case studies in infectious diseases

The gut microbiota’s metabolic activity influences host gene expression, contributing to diseases like type 2 diabetes or obesity.^[6]^ For instance, disturbances in bacterial microbiota result in dysregulation of adaptive immune cells, which may underlie disorders like IBD.^[74]^ The interplay between commensals and pathogens or indigenous pathobionts is critical for controlling infection and disease. Understanding pathogen–commensal interactions may lead to new therapeutic approaches.^[87]^ Additionally, gut microbiota-derived butyrate enhances colonic regulatory T-cell differentiation through its epigenetic modulatory ability via histone deacetylase inhibition, impacting immune regulation and diseases.^[88]^

### 4.16. Therapeutic implications in infectious diseases

Epigenetic therapies offer new possibilities in infectious disease treatment. These therapies, targeting DNA methylation and histone modifications, are pivotal in the pathogenesis of diverse infections. By focusing on these epigenetic alterations, new avenues of treatment emerge, especially valuable in scenarios where conventional antimicrobial methods falter or elicit resistance.^[89,90]^ Exploring synergistic opportunities between microbiota modulation and epigenetic therapies in infectious diseases is a burgeoning field. This innovative strategy incorporates microbiota-influencing interventions like targeted probiotics or dietary alterations alongside epigenetic medications. This dual approach bolsters the body’s defense against infectious agents, potentially elevating treatment efficacy.^[59,91]^ Adopting personalized medicine, attuned to individuals’ unique microbiota and epigenetic profiles, holds significant promise in infectious diseases. Such a tailored approach promises enhanced effectiveness by considering the distinct interaction between an individual’s microbiota and epigenetic factors in response to specific pathogens.

## 5. Public health implications

### 5.1. Screening and risk assessment

Microbiome and epigenetic profiles are valuable public health screening and risk assessment biomarkers. They can help identify individuals at higher risk for certain diseases, providing tools for early intervention and personalized prevention strategies.^[92]^ Gut microbiota dysbiosis profiles, for example, can serve as risk predictors for IBD or irritable bowel syndrome in preclinical cases. Similarly, gut microbiota profiles with dominant groups like firmicutes correlate with differential methylation status of gene promoters linked to cardiovascular diseases, lipid metabolism, and obesity, offering the potential for early disease detection and management.^[93]^

### 5.2. Public health strategies and interventions

Recent explorations have illuminated the complex interplay between diet, lifestyle, and the gut microbiome’s influence on epigenetics, underscoring their pivotal role in shaping public health. The critical function of dietary bioactivity, encompassing the constituents of the gut microbiome, is garnering recognition for its influential role in the emergence of diseases such as metabolic syndrome, immunoallergic diseases, obesity, and diabetes.^[94–96]^ Additionally, the beneficial alteration of the gut microbiota composition through probiotics, prebiotics, and dietary fiber is increasingly acknowledged.^[97]^ This understanding guide tailored dietary interventions, especially targeting the gut–brain axis in neurodevelopmental disorders.^[98]^ The profound influence of environmental determinants on the gut microbiome and epigenetic mechanisms calls for implementing policy measures conducive to healthy dietary practices and mitigating harmful toxin exposure. This policy direction holds substantial potential in mental health arenas, considering the pivotal role of gut microbiota in modulating mood and behavioral patterns.

### 5.3. Prevention and management approaches

Dietary influences are crucial in shaping the gut microbiome, impacting epigenetic modifications associated with conditions such as IBD. Including specific micronutrients and adopting anti-inflammatory dietary patterns have been demonstrated to offer potential benefits in preventing and managing such diseases.^[99,100]^ These findings underscore the importance of these dietary components in modulating disease pathways. Implementing community-based programs and educational campaigns emphasizing the significance of diet, lifestyle, and environmental factors is a promising strategy to enhance gut and epigenetic health. Such initiatives can exploit the growing understanding of dietary interactions with the intestinal mucosa, mainly how these interactions regulate immune homeostasis and affect the etiopathogenesis of diseases like IBD.^[91,101]^ This approach is supported by research highlighting the transformative potential of community-driven health interventions.

### 5.4. Pharmacological and probiotic therapies

Pharmacological and probiotic therapies, significant in public health, modulate gut microbiota and epigenetic states, pivotal for disease prevention and management. Strategies encompassing probiotics, prebiotics, antimicrobial agents, and bariatric surgery show potential in metabolic disease management through gut microbiota modulation.^[102]^ Probiotics, garnering interest for preventing and treating various disorders, including metabolic dysfunctions, inflammation, and bacterial infections, leverage gut microbiota modulation. This approach promises health enhancement and disease combat.^[97,103]^

### 5.5. Emerging research areas

In the evolving landscape of gut microbiota and epigenetics research, their comprehensive roles in health sustenance and disease prevention are becoming increasingly evident. The dynamic interaction between gut microbiota and epigenetics is scrutinized for its involvement in chronic ailments such as obesity, diabetes, and various immune-mediated disorders. These research domains might pave the way for innovative disease prevention and health enhancement strategies. For example, insights into how gut microbiota influences epigenetic mechanisms could inform the crafting of specific dietary interventions and lifestyle alterations to thwart or manage chronic conditions.^[104]^

### 5.6. Policy and healthcare system implications

The strides in gut microbiota and epigenetic research carry profound implications for healthcare policy. They ignite critical discussions about genetic versus environmental influences, the increasing social focus on biology, and the necessity for public health strategies that integrate these emerging insights.^[105]^ Policymaking might need evolution or modification to bolster research and its application in these spheres, especially concerning personalized medicine. Adapting healthcare systems to include gut microbiota and epigenetic findings in clinical practice is crucial. This integration could manifest in including microbiome and epigenome analyses in standard health evaluations and the inception of novel public health initiatives aimed at modulating these elements for disease prevention and management.^[106,107]^

## 6. Challenges and limitations

### 6.1. Complexity of interdisciplinary research

Fusing disciplines such as microbiology, genetics, epigenetics, and public health introduces formidable challenges in contemporary research landscapes. This integration, critical for identifying human disease risk factors and forging effective public health strategies, necessitates navigating barriers in communication, methodology, and data interpretation. The intricacy of amalgamating insights from diverse fields, notably epigenetics and public health genomics, intensifies with the hurdles in knowledge synthesis and translation, pivotal for converting genomic research into actionable policies and guidelines.^[108,109]^ This convergence is imperative for tackling complex health challenges.^[110]^

### 6.2. Methodological challenges

The methodological challenges in gut microbiota and epigenetics research are considerable and manifold. A primary concern is collecting and preserving samples for gut microbiome studies, where factors like sample quality, applicable omics techniques, user experience, and cost efficiency are pivotal. These factors are essential for maintaining sample integrity and representativeness, underlining the necessity for effective methods to safeguard these aspects.^[111]^ Moreover, the absence of universally acknowledged standards for gut microbiota research compounds the complexity. The diversity of protocols for sample collection and DNA extraction leads to result variability.^[112]^ Additionally, the interplay between gut microbiota and epigenetic regulation, influencing host health and disease, introduces an additional layer of intricacy. This interconnection has profound implications for therapeutic strategies, accentuating the importance of methodological precision.^[5]^ Establishing a protocol for self-collection of microbiome samples addresses some challenges.^[113]^ Nonetheless, biases in gut microbiota studies stemming from preservation solutions and bacterial 16S rRNA gene primer pairs underscore the need for consistent methodologies.^[114]^

### 6.3. Data interpretation and integration

The interpretation of data from microbiome and epigenome studies, inherently complex due to the vast and intricate nature of the information, presents substantial challenges. Epigenome-wide association studies unveil novel opportunities yet pose difficulties in design, cohort and sample selection, statistical significance, power, confounding factors, and the need for follow-up studies.^[115]^ Moreover, integrating high-dimensional genomic data, including microbiome data, necessitates advanced statistical methodologies like mediation analysis, high-dimensional instrumental variable regression, sparse signal recovery, and compositional data regression.^[116]^ This integration is vital for unraveling complex phenotypes and disease prevention, yet it has hurdles. Genetic variants associated with diseases and traits are found to be enriched in tissue-specific epigenomic marks, elucidating biologically pertinent cell types for various human traits and aiding in deciphering the molecular underpinnings of human diseases.^[117]^ The fusion of gene expression data with common risk variation and the incorporation of epigenome data enhances the precision of transcriptome predictions. It amplifies the ability to detect significant expression-trait associations.^[118]^ Techniques like functional linkage analysis and DNA methylation quantitative trait loci are instrumental in pinpointing functional genetic variability and refining interpretation strategies for risk genotypes and physiological traits.^[119]^ Nevertheless, translating these insights to human health is a formidable task, demanding meticulous consideration and methodological exactitude.

### 6.4. Causal relationships and translational research

In the field, a pivotal challenge is establishing definitive causal links between gut microbiota, epigenetic changes, and specific health outcomes. Mendelian randomization studies, incorporating human genetics, advance our understanding of causality in gut microbiome research. This approach is critical to crafting targeted therapies for enhanced health.^[120]^ However, the complexity lies in tracking gut dysbiosis grading to understand host phenotype-genotype associations and microbiota shifts, especially in deploying gut dysbiosis profiles as risk predictors for diseases like IBD or irritable bowel syndrome in preclinical scenarios.^[121]^ Transitioning laboratory discoveries to clinical applications remains a significant barrier. Emerging cultivation methods and high-throughput phylogenetic mapping propel studies of gut microbiota xenobiotic tolerance and modification phenotypes, potentially hastening the development of probiotics and other therapeutic measures.^[122]^ Despite these advancements, the connection of the human gut microbiome to diverse health outcomes continues to pose challenges in establishing causal relationships and translating these discoveries into efficacious clinical applications.^[123,124]^

### 6.5. Generalizability and reproducibility

Extending findings across diverse populations and environmental conditions presents a formidable challenge in gut microbiota and epigenetics research. Variations in the gut microbiome, attributed to factors such as the nature and source of biospecimens, DNA extraction techniques, handling environments, and bioinformatics methodologies, obstruct the universal applicability of findings.^[125]^ This variation leads to outcome discrepancies, complicating efforts to extend conclusions broadly. The interplay between gut microbiota and epigenetic mechanisms in conditions like IBD further complicates this scenario, as environmental influences and genetic predispositions play critical roles.^[126]^ Methodological divergences, such as microbial community sequencing, create hurdles in replicating studies.^[125]^ The impact of gut microbiota on epigenetic modifications, particularly DNA methylation and histone changes, varies considerably. This variance influences the consistency of results across different studies.^[59]^ These challenges underscore the necessity for expansive and varied research efforts, coupled with establishing uniform methodologies to enhance the validity and consistency of scientific conclusions. The nuanced interplay between gut microbiota and epigenetics demands a collaborative approach, uniting the fields of microbiology, genetics, epigenetics, immunology, and public health. The significant influence of substances originating from gut microbiota on epigenomic processes accentuates the need for cross-disciplinary cooperation.^[127]^ Embracing a systems biology view is fundamental to unraveling the shift from a healthy symbiotic state to a microbial imbalance in disorders related to the gut.^[128]^ The complex interaction between gut microbiota and epigenetics in sustaining health and thwarting disease further underscores the criticality of joint research efforts, especially in deciphering the impact of gut microbiota on immune system development and function.^[56]^

## 7. Conclusion

In the intricate realm of human health and disease, the profound interplay between gut microbiota and epigenetic mechanisms assumes a pivotal role as a conductor. Governed by a myriad of influencers like dietary cues, genetics, and environmental stimuli, this symbiotic relationship adeptly regulates nutrient metabolism, intricate immune responses, and defenses against pathogens. The groundbreaking advancements in genomics and metabolomics have unveiled profound correlations between these microbial orchestrators and the host’s physiological symphony, resonating across a spectrum of diverse diseases. Notably, the interaction between epigenetic regulators, such as DNA methylation and histone modifications, and the dynamic performance of the gut microbiota stands out, particularly observed in conditions like IBD and obesity. Addressing the complexities of data interpretation and methodological limitations emphasizes the urgent necessity for a multidisciplinary collaboration in this scientific pursuit. The integration of insights from microbiome and epigenome studies is not merely advantageous but imperative. This amalgamation holds the promise of illuminating the intricate pathways governing these interactions, potentially marking the advent of a new era in therapeutic innovations and public health strategies aimed at disease management and prevention.

## 8. Future research directions

While a lot can be said regarding the association of gut microbiota with epigenetic alterations, up to now, the exact molecular mechanism by which microbiota-derived metabolites influence DNA methylation and histone modifications remains to be elucidated. Future research has to identify exactly what pathways mediate these effects. Short-chain fatty acids, such as butyrate, propionate, and acetate, produced by the gut microbiota, are generally involved in potentially modulating epigenetic markers. For example, butyrate is a histone deacetylase inhibitor, which may lead to up- or downregulation of gene expression related to inflammation, cell proliferation, and apoptosis. In general, more profound studies are required to evaluate the extent of the effects of these SCFAs on epigenetic markers in different tissues and health outcomes. More importantly, other microbial metabolites, such as bile acids and vitamins, can be unraveled in their role in epigenetic regulation for a comprehensive understanding of their functions. Of note is the need to further the research for identifying specific microbial species most influential in modulating the epigenetic changes. For example, the study may focus on the function of bacteria such as *Faecalibacterium prausnitzii*, which owns anti-inflammatory properties, and *Bacteroides fragilis*, which is implicated in the modulation of the immune system. Understanding the specific interactions of these microbes and host epigenetic mechanisms may direct a way toward targeted probiotic therapies that reduce diseases such as IBD, obesity, and colorectal cancer. Further research in the study regarding other epigenetic modifiers, for instance, noncoding RNAs, and how they might respond to the influence of the gut microbiota. Noncoding RNAs, which include miRNAs and lncRNAs, are essential in gene regulation. Future studies should focus on how the gut microbiome can induce changes in the expression levels of these noncoding RNAs, thus allowing expression patterns of genes to be associated with health and disease.

## Author contributions

**Conceptualization:** Bailee Kim, Angel Song, Andrew Son, Yonghwan Shin.

**Writing – original draft:** Bailee Kim, Angel Song, Andrew Son.

**Writing – review & editing:** Yonghwan Shin.
